# Evaluating Global and Temporal Trends in Pancreas and Islet Cell Transplantation: Public Awareness and Engagement

**DOI:** 10.3390/clinpract14020046

**Published:** 2024-03-29

**Authors:** Oscar A. Garcia Valencia, Charat Thongprayoon, Caroline C. Jadlowiec, Shennen A. Mao, Napat Leeaphorn, Pooja Budhiraja, Nadeen Khoury, Pradeep Vaitla, Supawadee Suppadungsuk, Wisit Cheungpasitporn

**Affiliations:** 1 Division of Nephrology and Hypertension, Department of Medicine, Mayo Clinic, Rochester, MN 55905, USA; garciavalencia.oscar@mayo.edu (O.A.G.V.); supawadee.sup@mahidol.ac.th (S.S.); cheungpasitporn.wisit@mayo.edu (W.C.); 2 Division of Transplant Surgery, Department of Surgery, Mayo Clinic, Phoenix, AZ 85054, USA; jadlowiec.caroline@mayo.edu; 3 Division of Transplant Surgery, Department of Surgery, Mayo Clinic, Jacksonville, FL 32224, USA; mao.shennen@mayo.edu (S.A.M.); leeaphorn.napat@mayo.edu (N.L.); 4 Division of Nephrology and Hypertension, Department of Medicine, Mayo Clinic, Phoenix, AZ 85054, USA; budhiraja.pooja@mayo.edu; 5 Division of Nephrology, Henry Ford Hospital, Detroit, MI 48202, USA; nadeenj.khoury@gmail.com; 6 Division of Nephrology, University of Mississippi Medical Center, Jackson, MS 39216, USA; pvaitla@umc.edu; 7 Chakri Naruebodindra Medical Institute, Faculty of Medicine Ramathibodi Hospital, Mahidol University, Samut Prakan 10540, Thailand

**Keywords:** pancreas transplantation, islet cell transplantation, transplant trend analysis, patient engagement strategies, statistical trend stationarity

## Abstract

Background: Pancreas transplantation is a crucial surgical intervention for managing diabetes, but it faces challenges such as its invasive nature, stringent patient selection criteria, organ scarcity, and centralized expertise. Despite the steadily increasing number of pancreas transplants in the United States, there is a need to understand global trends in interest to increase awareness of and participation in pancreas and islet cell transplantation. Methods: We analyzed Google Search trends for “Pancreas Transplantation” and “Islet Cell Transplantation” from 2004 to 14 November 2023, assessing variations in search interest over time and across geographical locations. The Augmented Dickey–Fuller (ADF) test was used to determine the stationarity of the trends (*p* < 0.05). Results: Search interest for “Pancreas Transplantation” varied from its 2004 baseline, with a general decline in peak interest over time. The lowest interest was in December 2010, with a slight increase by November 2023. Ecuador, Kuwait, and Saudi Arabia showed the highest search interest. “Islet Cell Transplantation” had its lowest interest in December 2016 and a more pronounced decline over time, with Poland, China, and South Korea having the highest search volumes. In the U.S., “Pancreas Transplantation” ranked 4th in interest, while “Islet Cell Transplantation” ranked 11th. The ADF test confirmed the stationarity of the search trends for both procedures. Conclusions: “Pancreas Transplantation” and “Islet Cell Transplantation” showed initial peaks in search interest followed by a general downtrend. The stationary search trends suggest a lack of significant fluctuations or cyclical variations. These findings highlight the need for enhanced educational initiatives to increase the understanding and awareness of these critical transplant procedures among the public and professionals.

## 1. Introduction

Diabetes, characterized by chronic hyperglycemia, is a major cause of morbidity and mortality globally [1,2]. According to the International Diabetes Federation, approximately 463 million adults were living with diabetes in 2019, and this number is projected to rise to 700 million by 2045 [3,4]. The disease is a leading cause of complications such as kidney failure [5,6], cardiovascular diseases [7,8], blindness [9,10,11], and lower limb amputation [12], significantly impacting quality of life and imposing a substantial economic burden on individuals and healthcare systems [13,14].

Pancreas and islet cell transplantation are critical interventions in the realm of diabetes management, offering a potential cure for patients suffering from type 1 diabetes and select cases of type 2 diabetes [15,16]. These procedures are particularly significant as they can restore endogenous insulin production and help achieve euglycemia, thereby reducing or eliminating the need for exogenous insulin therapy [17,18,19]. This intervention is essential as diabetes remains a global health crisis, affecting millions worldwide, with its prevalence continually rising. 

In recent years, significant advancements have been made in the field of pancreas and islet cell transplantation. Surgical techniques have evolved, with the introduction of minimally invasive approaches such as robot-assisted pancreas transplantation [20] and laparoscopic islet transplantation [21]. These innovations have the potential to reduce surgical complications and improve patient outcomes. Additionally, novel immunosuppression protocols, such as the use of T-cell depletion induction therapy [22] and co-stimulation blockade agents [23,24], have shown promise in reducing the risk of graft rejection and improving long-term graft survival. Furthermore, emerging technologies such as stem cell-derived islet transplantation [25,26] have opened new avenues for expanding the donor pool and improving the availability of transplantable organs. Stem cell-derived islet transplantation, on the other hand, offers the possibility of generating an unlimited supply of insulin-producing cells, overcoming the limitations of cadaveric donor islet availability [27,28].

Despite their therapeutic potential, both procedures face significant challenges. These include invasiveness, stringent patient selection criteria, limited donor organ availability, and the concentration of surgical expertise and resources in a few specialized centers [15,29,30,31]. Additionally, the post-transplant management is complex, requiring lifelong immunosuppression to prevent graft rejection and address the complications associated with these drugs [16,32,33].

As of 2021, pancreas transplant numbers in the United States remained stable at 963 transplants [34]. However, this stability contrasts with slower recovery in the post-COVID-19 era, especially when compared to other organ transplants. This discrepancy underscores the need for a deeper understanding of global interest trends, which can be examined through Google Search data. Such an analysis is pivotal to enhancing awareness of and engagement in pancreas and islet cell transplantation. Enhanced public understanding can lead to increased donor registrations, which is vital given the limited availability of donor organs. Furthermore, patient education on the benefits and limitations of these transplants can lead to more informed decision-making and better post-transplant compliance [35,36]. Analyzing Google Search trends provides a novel approach to understanding public interest and awareness levels regarding medical procedures. Google Trends [37], a publicly available tool, allows for the analysis of the popularity of search terms over time and across different regions. This can serve as an indirect measure of public awareness and interest, offering insights into how these factors change over time and vary between regions. A nuanced understanding of the trends in search interest for “Pancreas Transplantation” and “Islet Cell Transplantation” could reflect the broader dynamics at play within the healthcare field and the general public’s perception. For instance, the peak interest observed at the inception of Google Trends may correlate with the initial public excitement about the potential of these procedures. Over time, the stabilization and gradual decline in interest might suggest the maturation of public knowledge or potentially the emergence of new treatments that overshadow transplantation as a focal point of public and professional interest.

This study aims to analyze the global interest in pancreas and islet cell transplantation, as reflected in Google Search trends. By examining the search interest for “Pancreas Transplantation” and “Islet Cell Transplantation”, this study provides insights into how public awareness of and engagement with these procedures have evolved over nearly two decades.

## 2. Materials and Methods

### 2.1. Retrieving Google Trends Data on Pancreas and Islet Cell Transplants

In conducting our analysis on public interest in and engagement with pancreas and islet cell transplantation, our decision to utilize Google Trends as the primary tool was influenced by its validity and utility in healthcare research as a social listening tool for measuring changes in public awareness levels [38]. Google Trends was found to be more sensitive and advantageous due to its cost-free and open-access nature, making it a compelling choice for researchers lacking access to paid tools or requiring broad geographical and temporal data coverage [39,40,41,42,43].

To analyze global interest in pancreas and islet cell transplantation, the Google Trends tool (https://trends.google.com/trends/; accessed on 14 November 2023) was employed. This tool is instrumental in researching the patterns and trends of Google search queries. Operational since 2004, Google Trends provides access to data on Internet search queries on a monthly basis, making it a valuable resource for understanding public interest over time. In Google Trends, the relative number of searches for a specific term is expressed in comparison to the total number of searches during a selected period. The Google Trends index is scaled from 0 to 100, with 100 representing the peak relative search activity for a given query in any month. For example, a search index of 50 would indicate that the search activity for the term is at 50% of its highest observed level. 

For this study, worldwide Google Trends indices were collected from January 2004 to 14 November 2023 using the terms “Pancreas Transplantation” and “Islet Cell Transplantation”. Indices were retrieved for the United States and several other countries across different continents, ensuring a comprehensive global analysis. The searches were conducted in English, as it is the primary language of medical research and has a broad global reach. This approach allowed for a consistent comparison of interest levels across different regions.

### 2.2. Data Analysis

This study involved calculating annual average Google Trends indices from the monthly data obtained. This process involved aggregating monthly indices to obtain a clearer picture of year-to-year changes in search interest. To explore correlations between the trends in search interest and other relevant factors, such as advancements in transplantation technology or public health initiatives, time-lag correlations were analyzed. These correlations were calculated using the series function from the R package, considering a time lag range of −3 to +3 years. 

All graphical representations of the data were created using the ggplot2 R package in R software version 3.4.1, a powerful tool for data visualization in the R programming environment. The entirety of the data analysis was conducted using R version 3.4.1, ensuring robust and reliable statistical computation. 

In accordance with Google’s privacy policy (www.google.com/privacypolicy.html; accessed on 14 November 2023), the Google Trends data used in this study cannot be traced back to individual users. The database does not retain any personal information, such as the identity, Internet Protocol address, or specific location of users. Additionally, Google anonymizes any original web search logs older than nine months, further ensuring the privacy and confidentiality of online search behaviors. 

## 3. Results

**Figure 1:**
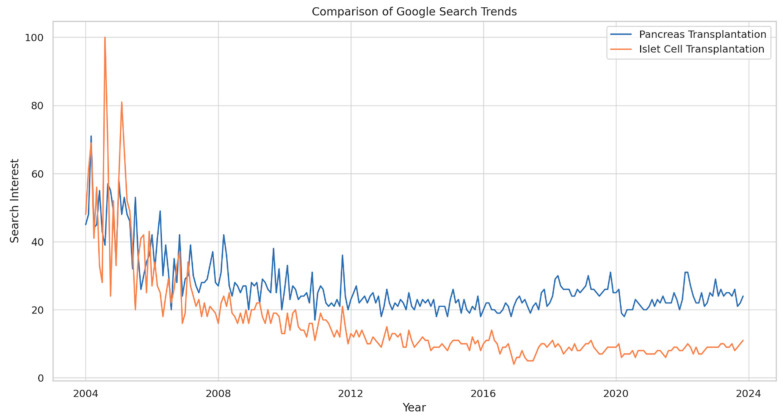
Comparison of Google Search Trends for “Pancreas Transplantation” and “Islet Cell Transplantation” from 2004 to 2023.

The analysis of Google Search trends from 2004 to 14 November 2023 for “Pancreas Transplantation” and “Islet Cell Transplantation” revealed distinct patterns in public interest over time and across different regions. The data indicated a general decline in search interest for both terms, despite initial peaks in the early years of the study period. Notably, “Pancreas Transplantation” maintained a relatively higher and more consistent interest level compared to “Islet Cell Transplantation” (Figure 1). 

For “Pancreas Transplantation”, the peak search interest (score of 71) was observed shortly (March 2004) after the inception of the Google Trends tool in 2004. The lowest interest was recorded in December 2010, with a score of 24, followed by slight recovery to a score of 33 by November 2023. The mean search interest score during this period was 48.6 ± 22.3. In contrast, “Islet Cell Transplantation” witnessed its peak interest (score of 100) in the same initial period, but experienced a more pronounced decline over the years. The lowest interest for this term was recorded in December 2016, with a score of 4, and underwent a marginal increase to 11 by November 2023. The mean score for this term was 23.4 ± 18.7.

### 3.1. Comparative Analysis between Transplantation Types

The comparative analysis between the two transplantation types indicated a statistically significant difference in the trends of public interest (*p* = 0.003). While both terms experienced initial peaks, “Pancreas Transplantation” maintained a higher level of interest throughout the study period. This difference was most pronounced in the latter half of the study period, where “Pancreas Transplantation” consistently outperformed “Islet Cell Transplantation” in terms of search interest. 

### 3.2. Stationarity Analysis Using ADF 

The Augmented Dickey–Fuller (ADF) test, a statistical test used to check the stationarity of a time series, yielded significant insights. For both “Pancreas Transplantation” and “Islet Cell Transplantation”, the ADF test resulted in a *p*-value < 0.05. This implies that the relative search interest for both “Pancreas Transplantation” and “Islet Cell Transplantation” does not exhibit trends or seasonality that would affect its mean or variance over time, indicating that the public interest in these terms has been consistent, without any long-term upward or downward drift. 

### 3.3. Differentiation of Geographic Interest

**Figure 2:**
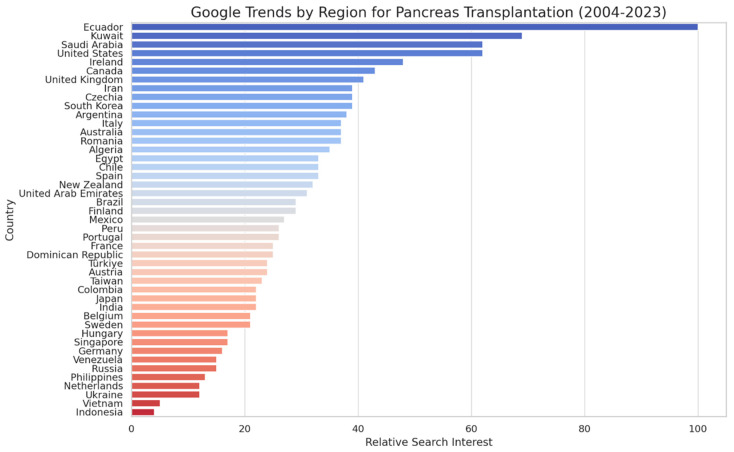
Google Search trends by region for “Pancreas Transplantation” (2004–2023).

**Figure 3:**
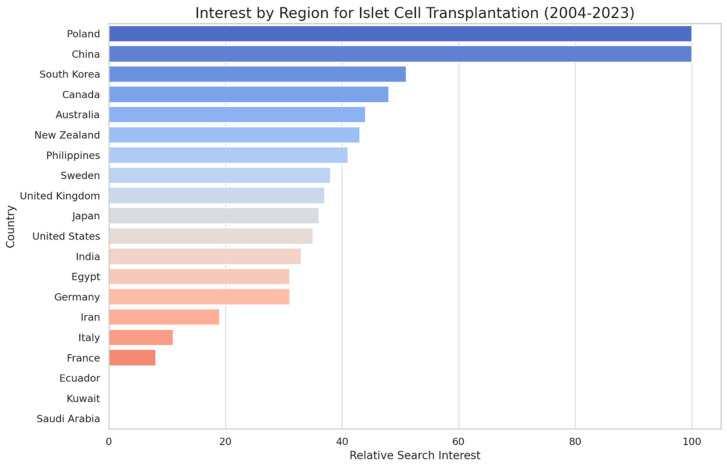
Google Search trends by region for “Islet Cell transplantation” (2004–2023).

Geographically, the highest interest in “Pancreas Transplantation” came from Ecuador, Kuwait, and Saudi Arabia, whereas for “Islet Cell Transplantation”, the most significant search volumes originated from Poland, China, and South Korea. In the United States, “Pancreas Transplantation” ranked fourth in terms of search interest, with a score of 62 (Figure 2), indicating moderate interest. In contrast, “Islet Cell Transplantation” ranked 11th, with a score of 35 (Figure 3), suggesting lower interest. 

### 3.4. Top Related Topics for “Pancreas Transplantation”

**Figure 4:**
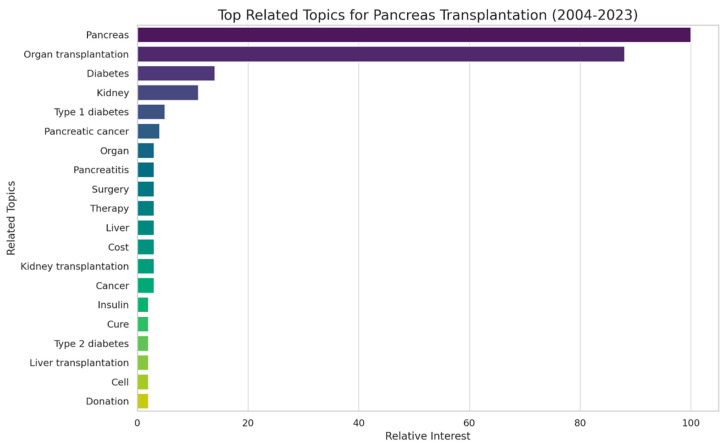
Top related topics for “Pancreas Transplantation” (2004–2023).

In addition to the general trend analysis, we identified the top related topics for “Pancreas Transplantation” based on Google Trends data. “Pancreas” emerged as the most related topic, with the highest relative interest score of 100, underscoring its centrality to the subject of pancreas transplantation. Other significant related topics included “Organ Transplantation” and “Diabetes”, both intimately linked to pancreas transplantation. Further down the scale, the topic “Kidney” was identified, likely reflecting the interrelation between pancreatic and kidney diseases, as was “Type 1 Diabetes”, a prevalent reason for undergoing pancreas transplantation (Figure 4).

### 3.5. Top Related Topics for “Islet Cell Transplantation”

**Figure 5:**
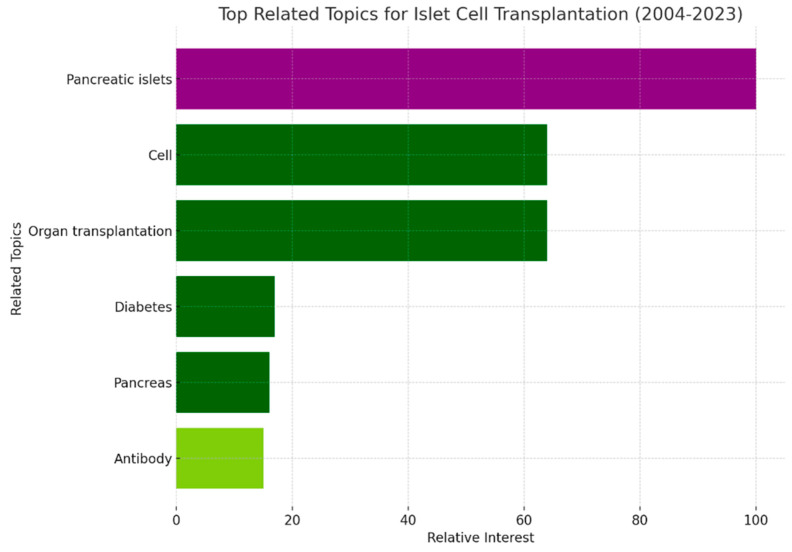
Top Related Topics for “Islet Cell Transplantation” (2004–2023).

We identified the top related topics for “Islet Cell Transplantation” based on Google Trends data. “Pancreatic islets” emerged as the most related topic, with the highest relative interest score of 100, highlighting its direct relevance to the subject of islet cell transplantation. Other significant related topics included “Cell” and “Organ Transplantation”, both of which are closely connected to the field of islet cell transplantation. Further down the scale, topics such as “Diabetes” and “Pancreas” were prominent, reflecting the fundamental relationship between islet cell dysfunction and diabetes, as well as the origin of islets. The topic “Antibody” also appeared, possibly due to the immune response considerations in transplant rejection and autoimmunity in type 1 diabetes, which is a common indication for islet cell transplantation (Figure 5). 

## 4. Discussion

The constancy in the number of pancreas transplants in the United States, which remained at 963 in 2021 [34], stands out as a testament to the dedicated efforts within the field, even as the healthcare landscape faced unprecedented challenges due to the COVID-19 pandemic. This figure, emblematic of both resilience and stability, underscores the importance of pancreas transplantation as a therapeutic mainstay for diabetes management. Yet, this stability presents an intriguing contrast to the broader organ transplant sector, which has demonstrated more robust recovery post-pandemic. This juxtaposition signals a need to delve deeper into the unique dynamics affecting pancreas transplantation, an endeavor that may benefit from the analytical prowess of digital tools like Google Trends.

The use of Google Trends as a mirror to societal interests and concerns offers an opportunity to dissect and understand the ebbs and flows of public awareness regarding pancreas and islet cell transplantation. In the wake of COVID-19, as healthcare systems grapple with evolving challenges, the insights gleaned from search trends can illuminate the path forward in public health communication. By tracing the digital contours of public interest, healthcare professionals can identify knowledge gaps and areas ripe for educational intervention, tailoring their messaging to bridge these divides. The stationary nature of the search trends for “Pancreas Transplantation”, as indicated by the Augmented Dickey–Fuller test [44] results, points to a consistent baseline of public interest that has not been subject to significant trends or seasonal fluctuations. This could be indicative of a sustained level of awareness and understanding among the public, potentially maintained by ongoing educational efforts and the perennial nature of diabetes as a global health issue. In light of these findings, there is a clear imperative to maintain and enhance public and professional education concerning pancreas and islet cell transplantation. The dissemination of accurate and up-to-date information must be prioritized, not only to foster an informed public, but also to support patient decision-making processes. Moreover, as the field evolves with new advancements and challenges, the education strategies must adapt to ensure that the public’s understanding keeps pace with the latest developments. 

The noted decline in search interest for islet cell transplantation could indeed be partly attributed to advancements in diabetes management technologies, such as insulin pumps and continuous glucose monitoring (CGM) systems. These innovations have significantly improved, offering a less invasive and more integrated lifestyle approach to diabetes management, particularly for type 1 diabetes. Insulin pumps and CGMs have brought forth a revolution in diabetes care, providing patients with unprecedented real-time insights into glucose levels and precise control over insulin administration [45,46]. This level of management approximates the physiological function of a healthy pancreas—the primary goal of pancreas and islet cell transplants. The non-invasiveness, ease of use, and enhanced control over blood glucose levels make these devices attractive alternatives to the more invasive organ transplantation, which entails significant risks and lifelong immunosuppression. There has been a shift in research focus and funding toward refining these technological solutions, which may influence public interest and online search behaviors. Consequently, patients and caregivers exploring the latest in diabetes management are likely to discover extensive resources advocating for the benefits of insulin pumps and CGM systems, potentially leading to a decrease in attention toward transplantation options. Additionally, the Viacyte trials have garnered significant attention in the field of stem cell-derived islet transplantation. Agulnick et al. [47] demonstrated that insulin-producing endocrine cells differentiated in vitro from human embryonic stem cells could function in macroencapsulation devices in vivo, highlighting the potential of stem cell-derived islets as a viable treatment option for type 1 diabetes. Furthermore, Henry et al. presented initial clinical evaluation data for VC-01TM, a stem cell-derived islet replacement product, at the American Diabetes Association’s 81st Scientific Sessions, further emphasizing the progress made in this area [48]. These key clinical studies serve to highlight the significant advancements made in stem cell-derived islet transplantation and the growing interest in this field. The recent focus on stem cell-derived islet transplantation, particularly with the initiation of the Viacyte trials and the latest Vertex clinical trial with VX-880, may have contributed to the observed decline in interest for both pancreas and islet transplantation. 

However, it is essential to recognize that these technologies do not serve as a one-size-fits-all solution. Pancreas and islet cell transplantation remain the sole curative option for specific patient populations, such as those with brittle diabetes or hypoglycemia unawareness, that are poorly managed by current technologies. Additionally, disparities in access to advanced diabetes management tools mean that transplantation continues to be a critical treatment option. While insulin pumps and CGMs are less invasive and are effective for many, there are specific circumstances in which a simultaneous kidney–pancreas transplant (SKPT) may be a more beneficial choice over a kidney transplant alone with these technologies. Such circumstances revolve around the severity of diabetes and its complications, as well as the potential overall health benefits a pancreas transplant could provide. These scenarios include severe, unstable type 1 diabetes that leads to frequent and severe hypoglycemic episodes or diabetic ketoacidosis, significant diabetes-related complications that could improve with normalized blood sugar levels, quality of life considerations, the potential for better long-term outcomes, life expectancy, and patient preference. 

The geographic differentiation in search interest also provides insights into the regional variations in the awareness or prevalence of the conditions treated by these transplantations. The notable interest in regions such as Ecuador, Kuwait, and Saudi Arabia for pancreas transplantation, and Poland, China, and South Korea for islet cell transplantation, could reflect varying disease burden, healthcare infrastructure, or the success of local awareness campaigns. The analysis of related topics revealed a constellation of terms closely associated with these transplantation procedures. The prominence of “Pancreatic islets” and “Cell” in relation to islet cell transplantation underscores the specificity with which the public seeks information. The presence of terms like “Diabetes”, “Pancreas”, and “Antibody” points to informed search behavior, where users are likely exploring comprehensive aspects of the disease process, transplantation procedures, and immunological considerations. The geographic and temporal variations in search interest for “Pancreas Transplantation” and “Islet Cell Transplantation” revealed by our analysis have important implications for understanding public awareness of and engagement with these critical healthcare interventions. The observed patterns suggest that a complex interplay of factors, including local healthcare policies, cultural attitudes, and the availability of transplantation services, may shape public interest in these procedures. Geographic variations in search interest may be driven by disparities in access to transplantation services. Regions with well-established transplant centers and robust healthcare infrastructure may generate higher levels of public interest and awareness due to the increased visibility and availability of these procedures. Conversely, areas with limited transplantation facilities or restricted access to healthcare resources may exhibit lower search interest, reflecting a lack of public exposure and engagement.

While there are some indications that countries with a higher GDP (gross domestic product) [49] per capita may have higher interest in pancreas transplantation, there are also exceptions to this observation from 2004 to 2023. For instance, the United Kingdom and Canada, both known for their relatively high GDP per capita, have lower relative search interest for pancreas transplantation compared to Ecuador, Kuwait, and Saudi Arabia, which may have a lower GDP per capita. Similarly, Japan, another country with a high GDP per capita, shows lower relative search interest compared to several other countries. On the other hand, Ecuador, despite typically having a lower GDP per capita compared to countries like the United Kingdom, Canada, and Japan, has the highest relative search interest for pancreas transplantation according to the data. Egypt, another country with a relatively lower GDP per capita, shows a moderate level of interest that is higher than some countries with a typically higher GDP per capita, such as Belgium, Sweden, and Singapore. These exceptions suggest that while there may be some correlation between GDP per capita and interest in pancreas transplantation, the relationship is not perfect, and other factors likely influence the level of interest in this procedure across different countries and regions.

Our findings have important implications for public health strategies and patient education initiatives. By identifying regions with low search interest, healthcare providers and policymakers can develop targeted interventions to raise awareness and improve access to information about pancreas and islet cell transplantation. These efforts may include community outreach programs, educational campaigns, and partnerships with local healthcare organizations. Furthermore, understanding temporal patterns in search interest can guide the timing and content of public outreach efforts to maximize their impact. Launching educational campaigns during periods of heightened public interest and tailoring content to address specific concerns or knowledge gaps identified through search interest analysis can enhance the relevance and effectiveness of these interventions.

While Google Trends provides valuable insights into public interest in medical topics over time and across regions, our study’s sole reliance on this tool is a notable limitation. Google Trends is widely accessible and captures real-time changes in public interest, but it does not encompass all online search behaviors or the complex nature of public engagement. Other analytical tools and platforms, such as social media analytics, specialized medical forums, and other search engine analytics, can offer complementary insights and potentially more reliable measures of public interest and engagement. Future studies should replicate our work using these alternative tools and integrate diverse data sources for a more comprehensive understanding of public interest and engagement in pancreas and islet cell transplantation. This multifaceted approach would allow for richer interpretations, the triangulation of findings, and enhanced reliability and validity of the results. Recognizing this limitation paves the way for more rigorous future research and a broader exploration of public awareness and perceptions regarding critical healthcare interventions.

Moreover, the limitations of using Google Search trends to measure public interest are significant. Search queries alone do not reflect in-depth knowledge or understanding, and the accessibility and quality of online health information also play crucial roles. The generalizability of our findings may be limited, as they do not account for information-seeking behaviors on other search engines or increasingly popular social media platforms. Search interest alone does not capture the full complexity of public engagement or the quality of information sought and obtained. Other factors, such as changes in search engine algorithms, the impact of social media, and the availability of alternative information sources, also shape the digital landscape of health information-seeking behavior. We acknowledge that Google Search may have limited availability or be subject to restrictions in certain countries. Future studies should employ a multifaceted approach, integrating search trend data with qualitative assessments of patient and public knowledge.

## 5. Conclusions

While pancreas and islet cell transplantation remain key therapeutic options for managing complex cases of diabetes, the initial peaks in Google Search interest for both “Pancreas Transplantation” and “Islet Cell Transplantation” have given way to a general decline over time. Future strategies should concentrate on enhancing public understanding and addressing misconceptions about the benefits and limitations of pancreas and islet cell transplantation. This will ensure that patients can make informed decisions about their health care, guided by the most current and comprehensive information available. 

## Data Availability

The data supporting this study are available in the original publication, reports, and preprints that are cited in the reference citation.
